# Conjunctival HLA-DR Expression and Its Association With Symptoms and Signs in the DREAM Study

**DOI:** 10.1167/tvst.8.4.31

**Published:** 2019-08-21

**Authors:** Neeta S. Roy, Yi Wei, Yinxi Yu, Gui-Shuang Ying, Eric Kuklinski, Brendan Barry, Maureen G. Maguire, Reza Dana, Mary Brightwell-Arnold, Penny A. Asbell, for the Dry Eye Assessment and Management (DREAM) Study Group

**Affiliations:** 1 Department of Ophthalmology, Hamilton Eye Institute, University of Tennessee Health Science Center, 930 Madison Avenue, Memphis, TN, USA; 2 Department of Ophthalmology, Perelman School of Medicine, University of Pennsylvania, 3535 Market Street, Suite 700, Philadelphia, PA, USA; 3 Department of Ophthalmology, Icahn School of Medicine at Mount Sinai, New York City, NY, USA; 4 Massachusetts Eye and Ear, Harvard Medical School, Boston, MA, USA

**Keywords:** HLA-DR, dry eye disease, conjunctival cells, symptoms and sign, correlation

## Abstract

**Purpose:**

Evaluation of dry eye disease (DED) relies on subjective symptoms and signs. We examined HLA-DR expression (HLA-DR%) in conjunctival cells, a minimally invasive biomarker with objective metrics, as an alternative method.

**Methods:**

Dry Eye Assessment and Management (DREAM) study participants completed the Ocular Surface Disease Index questionnaire. Clinicians evaluated tear volume, tear breakup time, and corneal and conjunctival staining. Conjunctival impression cytology samples (*n* = 1049) were assessed for HLA-DR% in total cells (TCs), epithelial cells (ECs), and white blood cells (WBCs). Associations (categorized into <5%, 5%–15%, >15%–25%, and >25%) with symptoms and signs were evaluated.

**Results:**

The HLA-DR% varied markedly across samples. Over 40% had <5 HLA-DR% positive cells in TCs and ECs and under 23% in WBCs. Higher HLA-DR% was associated with higher conjunctival staining for ECs (mean score 2.77 for <5% and 3.28 for >25%, linear trend *P* = 0.009) and TCs (mean score 2.82 for <5% and 3.29 for >25%, linear trend *P* = 0.04) and in TCs was associated with higher corneal staining (mean score 3.59 for <5% and 4.46 for >25%, linear trend *P* = 0.03). HLA-DR% in WBCs did not correlated with signs (all *P* ≥ 0.58), and in TCs, ECs or WBCs were not associated with symptoms (*P* > 0.06).

**Conclusions:**

The distribution of HLA-DR% in conjunctival cells reflects the heterogeneity of disease in DREAM participants. High percentages of samples with <5% positive cells indicate that HLA-DR% may not be a sensitive marker for DED in all patients.

**Translational Relevance:**

High HLA-DR% in ECs in association with high conjunctival staining may identify a subgroup of DED patients prone to epithelial disease and possibly need a different approach from current standards of treatment.

## Introduction

Dry eye disease **(**DED) is a common ocular condition with a worldwide prevalence ranging from 5% to 50%.^1^ As defined by the International Dry Eye Workshop in 2017:

Dry eye is a multifactorial disease of the tears and ocular surface, that results in symptoms of discomfort, visual disturbance, and tear film instability with potential damage to the ocular surface. It is accompanied by increased osmolarity of the tear film and inflammation of the ocular surface.^2^

DED is common with advancing age, may complicate eye surgeries, and is considered an important risk factor for severe corneal complications, which includes perforations, infection, neovascularization, and stem cell insufficiency. Clinically, the diagnosis of DED and the assessment of severity rely on symptoms from patient-reported questionnaires and signs based on clinical tests.^3^,^4^ However, due to the multifactorial nature of DED, correlation between symptoms and signs is poor and inconsistent.^5^–^8^ This has major implications on the development of reliable tools for diagnosis, classification, and measurement of treatment response.

Although the pathogenesis of DED is not fully understood, it is recognized that immune-mediated inflammation plays prominent roles in both development and progression.^9^–^13^ Consequently, several studies have looked at inflammation-related receptors and proteins to identify objectively quantifiable biomarkers to complement or even replace current DED assessment modalities.^14^–^17^ The major histocompatibility complex class II antigen HLA-DR is one of the most commonly studied inflammatory markers in DED.^18^–^34^ HLA-DR is a surface receptor that is constitutively expressed on antigen presenting cells, such as dendritic cells, monocytes, B cells, and activated T cells.^35^ Under pathological conditions, it can be conditionally induced in epithelial and other cells to regulate local immune responses.^36^,^37^ Multiple studies have looked at HLA-DR expression (HLA-DR%) in DED patients by using impression cytology (IC), a technique for sampling cells from the ocular surface, and analysis with flow cytometry.^38^,^39^ These studies have reported elevated HLA-DR% in DED patients and changes in HLA-DR% following different treatment regimens.^18^–^34^ However, the validity of HLA-DR as an objectively measurable parameter faces two issues. The first is the lack of universal standardization of the methodology for HLA-DR detection and measurement; studies have reported wide ranges of average baseline HLA-DR% in DED patients.^17^ The second is the limited and inconsistent correlation of HLA-DR% with commonly used clinical assessments of signs and symptoms.^23^,^30^,^34^ This second limitation is of particular importance in validating HLA-DR as a biomarker in DED.^40^,^41^

The purpose of the present study was to determine the characteristics of ocular surface HLA-DR% by using stringently established and verified standard operating procedures (SOPs)^39^ and to assess associations between ocular HLA-DR% levels and dry eye (DE) symptoms and signs in a well-characterized population of patients recruited for a multicenter, placebo-controlled, double-masked randomized clinical trial: The Dry Eye Assessment and Management (DREAM) study (Clinicaltrials.gov identifier NCT02128763).^42^

## Methods

### Participants and Study Design

Patients with moderate to severe DED were recruited and screened at 27 clinical centers throughout the United States. The study participants had moderate to severe DED symptoms as defined by Ocular Surface Disease Index (OSDI) ≥21.^43^ The major inclusion criteria were age (>18 years) and a minimum of 2 out of 4 of the following signs in at least one eye: conjunctival lissamine green staining present ≥ 1 out of a possible score of 6/eye (graded 0–3 in the nasal and temporal region, with 0 = no coloration, 1 = some punctations, 2 = well defined punctations, and 3 = many punctations), corneal fluorescein staining present ≥ 4 out of a possible score of 15 per eye (graded 0–3 in the central, top, bottom, temporal, and nasal sections of the eye), tear film break up time (TBUT) ≤ 7 seconds and Schirmer's test with anesthesia ≥1 to ≤7 mm/5 minutes (for comprehensive list see Ref. 44). Major exclusion criteria were contact lens wear, ocular surgery within 6 months of screen visit, use of glaucoma medication, and eyelid abnormalities that affect lid function (for comprehensive list see Ref. 44). In addition, participants needed to have had DE symptoms for at least 6 months and the desire to use artificial tears for an average of two times per day in the 2 weeks prior to screening visit. The study protocol was approved by institutional review boards associated with each center. Written informed consent was obtained prior to initiation of any study-related procedure. The study was conducted in accordance with the tenets of Declaration of Helsinki.

### Assessment of DE Symptoms and Signs

At baseline, patients underwent evaluation for multiple DED symptoms and signs. The OSDI questionnaire consisted of 12 questions with ratings from 0 to 4 with scoring to transform the responses to a 0 to 100 scale, with a higher score indicating more symptoms. TBUT was measured 30 seconds after instillation of 5 μL 2% fluorescein-containing solution. The time between the last blink and the appearance of the first discontinuity in the fluorescein-stained tear film was noted and repeated twice. Corneal fluorescein staining was graded using the cobalt blue filter of a slit lamp approximately 2.5 minutes after fluorescein instillation. Staining was scored using the National Eye Institute [NEI]/industry-recommended guidelines (0–15).^45^ Conjunctival staining was graded after 1 to 2 minutes of placing 5 μL of 1% lissamine green dye into the lower conjunctival sac. Grading was done for the nasal-bulbar and temporal-bulbar conjunctiva by using a modified version of the NEI/industry-recommended guidelines; the temporal and nasal section of each eye was graded on a scale of 0 to 3 (0 = no staining, 3 = severe staining) for a total possible score of 6 in each eye. Assessments for signs were done first in the right eye and then in the left eye. Schirmer's test was performed, following administration of preservative-free topical anesthetic, by inserting the strip at the junction of the lower lid for 5 minutes.

### Conjunctival Sample Collection

Conjunctival samples were collected from both eyes by IC by using an established SOP.^25^,^39^ Care was taken to ensure a lapse of 20 minutes between IC sampling and the last clinical test. In short, following anesthetization of both eyes with 0.5% proparacaine hydrochloride ophthalmic solution, one-half of a sterile 0.20-μm, 13-mm polyether sulfone filter membrane (Supor 100, 13-mm diameter, pore 2 μm; Gelman, Pall Sciences, Fort Washington, NY) was placed onto the superotemporal area and the other half on the inferotemporal area of the bulbar conjunctiva. Filters papers were then placed in tubes containing 2 mL of cold sterile phosphate-buffered saline with 0.01% paraformaldehyde. Samples from each eye were placed in separate tubes and analyzed separately (OD and OS). A drop of prophylactic antibiotic (0.5% moxifloxacin hydrochloride) was administered to patient eyes postcollection. The tubes were stored and shipped at 4°C and processed within 30 days of collection. All cell processing was done at the Ocular Biomarker Laboratory located at the Icahn School of Medicine at Mount Sinai, New York City, NY. All samples were labeled with both a numeric and an alphabetic identification code.

### Sample Processing

Cells were harvested from filter papers by shaking tubes at 400 rpm for 20 minutes at 4°C, followed by vortexing for 20 seconds. An additional 2 mL of sterile phosphate-buffered saline with 0.5% BSA (buffer) was added to each tube, and filter papers were removed and discarded. The tubes were then centrifuged at 1000 rpm for 10 minutes. Supernatant was aspirated, leaving behind 100 μL of liquid and 50 μL of antibody cocktail containing BV711-labeled anti-human HLA-DR antibody (Biolegend, San Diego, CA), Phycoerithrin-labeled anti-EpCAM (epithelial cell marker) antibody (BD Biosciences, Franklin Lakes, NJ), pacific orange-labeled anti-CD45 (pan-white blood cell marker) antibody (Invitrogen, Carlsbad, CA), and antibodies to other immune cell markers (not reported in this paper) were added to each tube, gently vortexed, and incubated at room temperature for 20 minutes. Antibody concentrations used in the cocktail for a particular antibody lot were based on prior antibody titrations performed with peripheral blood mononuclear cells to determine optimal signal-to-noise ratio. This minimized the noise from nonspecific binding of the antibodies to low-affinity targets and to achieve the brightest signal with the lowest background. Titrations were performed for each new antibody lot. Finally, the cells were washed with 2 mL of buffer by centrifuging for 10 minutes at 1000 rpm and resuspended in 170 μL of buffer for flow cytometry.

### Flow Cytometry, Controls, and Analysis

All samples were analyzed using a BD LSRFortessa cell analyzer. Each sample set in the study was analyzed using a common configuration established for the flow cytometer at the beginning of the study. For each sample run, eight peaked Rainbow Calibration Particles (BD Biosciences) were run to track cytometer performance and compensation was calculated using single antibody stained beads (BD CompBead) to eliminate spectral overlap associated with simultaneous usage of multiple fluorochrome-labeled antibodies. In addition, Fluorescence Minus One controls were periodically performed to ensure antibody binding specificity as well as to demarcate gating areas for positive cell populations. This required the preparation of multiple cocktails, each one missing one specific antibody from our panel and staining pooled conjunctival IC samples (from one individual) separately with each of these minus-one antibody cocktails. Flow cytometer outputs were imported and analyzed using the FCS Express 6 data analysis software (De Novo Software). All analysis was performed using a hierarchical gating strategy developed at Ocular Biomarker Laboratory. To reduce user-based variations in gating of cell populations, the two personnel conducting the analysis periodically analyzed and compared data with a common set of samples.

### Statistical Analysis

SAS v9.4 (SAS Institute, Inc., Cary, NC) was used for statistical analysis. Continuous measures were summarized using mean, standard deviation (SD), standard error (SE), median, and interquartile range. Categorical measures were summarized using percentage. Due to the very skewed distribution, the % of HLA-DR% in an eye was categorized into four levels (<5%, 5%–15%, >15%–25%, and >25% cells expressing HLA-DR), and the associations of HLA-DR% with DED symptoms and signs were evaluated using analysis of variance and a linear trend test that accounted for the intereye correlation by using generalized estimating equations. These analyses were performed for HLA-DR% in total cells (TCs), in epithelial cells (ECs), and white blood cells (WBCs). A value of two-sided *P* < 0.05 was considered to be statistically significant.

## Results

### Study Population

A total number of 535 patients with moderate to severe DED were recruited. Of these, data were analyzed for 527 patients because 7 patients had no IC samples collected and 1 had insufficient gated cells (<1000) for both eyes. The final data set represents 1049 IC samples (one per individual eye), as 1 eye each from 5 patients had to be excluded for having <1000 total gated cells.

### Demographic and Clinical Characteristics

Females constituted 81.2% (*n* = 428) of the total patients analyzed. Ages ranged from 18 to 87 years, with the median age being 60 years. The characteristics are summarized in Table 1. The mean (SD) OSDI score was 42 (15.5). The mean (SD) was 2.9 (1.5) for conjunctival staining score, 3.8 (3.0) for corneal staining score, 3.1 (1.7) seconds for TBUT time, and 9.6 (7.1) mm for Schirmer's test (Table 1). The intereye correlation was 0.67, 0.8, 0.65, and 0.80, respectively, for conjunctival staining, corneal staining, TBUT, and Schirmer's test.

**Table 1:** Baseline Characteristics of Patients Analyzed in the Study

Characteristics	Values (*N* = 527 Patients)
Age (years), mean ± SD	58.2 ± 13.1
Sex, *n* (%)	
Female	428 (81.2)
Male	99 (18.8)
Total OSDI score, mean ± SD	42.0 ± 15.5
	(*N* = 1049 eyes^a^)
Conjunctival staining score, mean ± SD	2.9 ± 1.5
Corneal staining score, mean ± SD	3.8 ± 3.0
Tear break-up time (sec), Mean ± SD	3.1 ± 1.7
Schirmer test (mm), mean ± SD	9.6 ± 7.1

a IC samples from only 1049 eyes were included in the analysis as 1 eye each from 5 patients had <1000 cells.

### Distribution of ECs and WBCs in Conjunctival IC Samples

**Figure 1:**
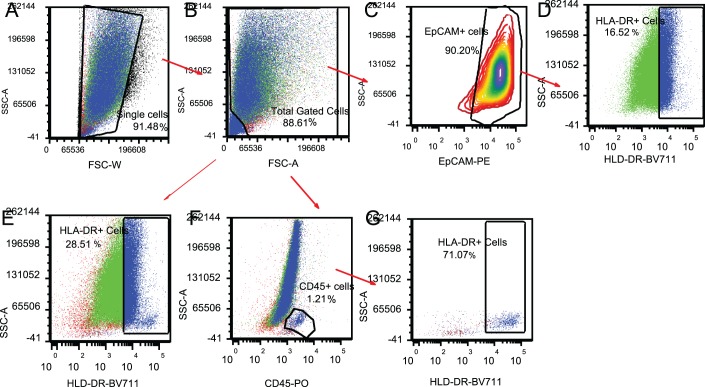
Flow cytometer analysis. Dot plots of a representative sample from the study. Total analyzable cells (88.61%) (B) were gated out from a scatter plot of FCS-W (Forward Scatter-Width) versus SSC-A (Side Scatter-Area; A). Sequential gating of EpCAM- or CD45-expressing cells from the total population yielded 90.20% ECs (C) and 1.21% WBCs (F). EpCAM+/HLA-DR+ cells (D) comprise 16.52% of the EC population and CD45+/HLA-DR+ cells (G) comprise 71.07% of the total WBCs.

The total number of single analyzable cells recovered from conjunctival IC samples per eye ranged from 1061 to 88,097 cells, with a median of 14,247 and mean ± SD of 15,507 ± 8673. Figure 1 shows the gating strategy used to demarcate the different cell populations on a representative sample. Of the gated TCs from 1049 conjunctival samples, ECs constituted 81.2% ± 13% (mean ± SD) and WBCs comprised 1.5% ± 1.7% (mean ± SD) with a range of 0% to 21.1% and a median of 1.1%.

### Distribution of HLA-DR% in Conjunctival Cell Populations

**Figure 2:**
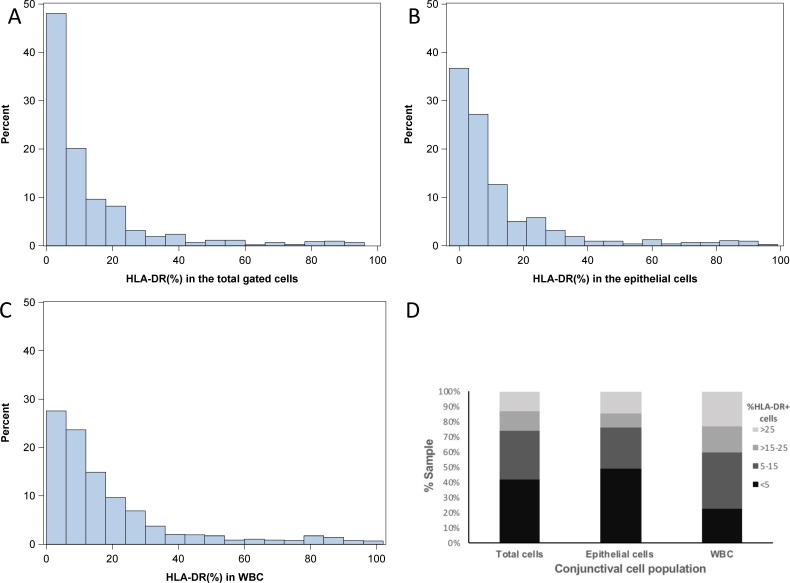
Distribution of HLA-DR in patient conjunctival cells. (A, B, and C) Show the percentage sample distribution (y axis) of HLA-DR% (x axis) in TCs (TCs), EpCAM-expressing ECs, and CD45-expressing WBCs, respectively, in the 1049 eye samples analyzed. (D) Stacked bars showing the percent distribution of samples in the four levels of HLA-DR% (<5%, 5%–15%, >15%–25%, and >25%).

There was a wide and skewed distribution in the percentage of cells expressing HLA-DR across the samples analyzed. (Figs. 2A, 2B, 2C). The median (interquartile range) of the % of HLA-DR% was 6.5 (2.6, 15.6) for TCs, 5.2 (1.8, 14.1) for ECs, and 11.6 (5.6, 23.5) for WBCs (Table 2). The intereye correlation for % of HLA-DR% was 0.81 in TCs, 0.84 in ECs, and 0.77 in WBCs. As some studies have excluded samples with <10,000 cells in their analysis due to concerns of data reproducibility with low cell numbers,^20^,^30^,^46^,^47^ we further analyzed the data for HLA-DR% by excluding samples consisting of <10,000 cells (30% of the samples). The median (interquartile) of HLA-DR% was 7.7 (3.1, 17.9) in TCs, 6.3 (2.5, 16.9) in ECs, and 10.0 (4.3, 22.2) in WBCs.

**Table 2:** Distribution of HLA-DR in TCs, ECs, and WBC (N = 1049 Eyes)

	TCs	ECs	WBCs
HLA-DR% as continuous			
Mean (SD)	13.0 (17.5)	12.5 (18.5)	19.0 (21.0)
Median (IQR)	6.5 (2.6, 15.6)	5.2 (1.8, 14.1)	11.6 (5.6, 23.5)
Range	(0.1–94.9)	(0.0–96.2)	(0.0–100.0)
HLA-DR% categories, *n* (%)			
<5% (negative)	442 (42.1)	515 (49.1)	239 (22.8)
5%–15% (low)	338 (32.2)	288 (27.5)	387 (36.9)
>15%–25% (middle)	129 (12.3)	98 (9.3)	185 (17.6)
>25% (high)	140 (13.3)	148 (14.1)	238 (22.7)

IQR, interquartile range.

Due to the wide variation in % of HLA-DR-expressing cells, cell populations were stratified into four levels of HLA-DR%, with <5% representing negative expression, 5% to 15% low expression, >15% to 25% medium expression, and >25% as the highest expression. Almost 50% of the samples showed <5% of HLA-DR% in ECs, while 42.1% and 22.8% of the samples showed <5% of HLA-DR% in TCs and WBCs, respectively. Table 2 and Figure 2D summarize the frequency distribution of sample percentages for each expression level and cell group.

### HLA-DR% Correlation With Demography, Symptoms, and Signs

Age and sex were not associated with HLA-DR% in TCs, ECs, or WBCs (Table 3). There was no significant association between HLA-DR in either TCs, ECs, or WBCs with DE symptoms regardless of whether HLA-DR% was divided into four groups based on expression levels (<5%, 5%–15%, >15%–25%, and >25%) or grouped by number of eyes (0, 1, 2) with positive (≥5%) HLA-DR% (Table 4).

**Table 3:** Association of Baseline HLA-DR% With Age and Sex

	*N*	Total Gated Cells^a^		ECs^a^		WBCs^a^	
		Median (IQR)	*P* Value	Median (IQR)	*P* Value	Median (IQR)	*P* Value
Age (years)			0.15^b^		0.13^b^		0.78^b^
18, 30	17	5.9 (1.9, 21.5)		4.6 (1.6, 22.7)		11.2 (5.1, 26.0)	
30, 40	33	5.0 (2.1, 11.4)		5.2 (1.4, 11.5)		12.2 (7.5, 19.6)	
40, 50	67	6.2 (2.8, 14.3)		6.1 (2.0, 15.7)		13.7 (6.8, 25.9)	
50, 60	137	6.0 (2.0, 15.6)		5.3 (1.7, 15.1)		11.8 (5.7, 20.4)	
60, 70	183	6.1 (2.8, 11.6)		5.7 (2.1, 11.4)		11.1 (5.9, 20.6)	
70, 80	72	8.0 (2.5, 18.3)		6.4 (2.2, 19.9)		13.8 (5.6, 22.6)	
≥80	18	25.0 (5.5, 82.4)		22.3 (5.0, 81.4)		26.5 (10.6, 68.1)	
Sex			0.26^c^		0.56^c^		0.88^c^
Female	428	6.1 (2.5, 14.4)		5.7 (2.0, 14.8)		12.0 (5.9, 21.7)	
Male	99	7.9 (2.2, 18.5)		6.0 (1.9, 18.0)		11.9 (6.6, 23.4)	

IQR, interquartile range.

a Using average of two eyes.

b From test of Spearman correlation coefficient.

c From Wilcoxon rank sum test.

**Table 4:** Baseline Association of HLA-DR% With OSDI at Eye Level and Person Level

Association With OSDI at Eye Level				Association With OSDI at Person Level			
HLA-DR	*N*	OSDI Mean (SE)	*P* Value^a^	Eyes Positive for HLA-DR	*N*	OSDI Mean (SE)	*P* Value
TCs			0.40	TCs			0.53
<5%	442	42.3 (1.0)		0	172	41.8 (1.2)	
5%–15%	338	43.0 (1.0)		1	103	44.6 (1.5)	
>15%–25%	129	40.2 (1.7)		2	252	41.0 (1.0)	
>25%	140	39.8 (1.6)					
ECs			0.06	ECs			0.58
<5%	515	42.2 (0.9)		0	209	41.9 (1.1)	
5%–15%	288	43.3 (1.0)		1	102	44.0 (1.5)	
>15%–25%	98	41.6 (1.9)		2	216	41.1 (1.1)	
>25%	148	38.4 (1.4)					
WBCs			0.23	WBCs			0.18
<5%	239	43.2 (1.2)		0	69	43.2 (1.9)	
5%–15%	387	41.3 (1.0)		1	106	43.6 (1.5)	
>15%–25%	185	42.3 (1.4)		2	352	41.3 (0.8)	
>25%	238	41.4 (1.3)					

a From test of linear trend.

Among the four assessed signs (Table 5), higher % of HLA-DR in ECs was associated with higher scores of conjunctival staining (mean score 2.77 for <5% and 3.28 for >25% linear trend *P* = 0.009). Higher HLA-DR% in TCs was associated with higher score of both conjunctival staining (mean score 2.82 for <5% and 3.29 for >25% linear trend *P* = 0.04) and corneal staining (mean score 3.59 for <5% and 4.46 for >25%, linear trend *P* = 0.03). No significant correlation was observed between the four DE signs and HLA-DR% in WBCs (Table 5).

**Table 5:** Association of Baseline HLA-DR With Dry Eye Symptoms and Signs at Eye Level

HLA-DR%	*N*	Conjunctiva Staining Score		Corneal Staining Score		Tear Break-Up Time (sec)		Schirmer Test (mm)	
		Mean (SE)	*P* Value^a^	Mean (SE)	*P* Value^a^	Mean (SE)	*P* Value	Mean (SE)	*P* Value^a^
TCs			0.04		0.03		0.68		0.48
<5%	442	2.82 (0.09)		3.59 (0.17)		3.08 (0.09)		9.43 (0.42)	
5%–15%	338	2.91 (0.09)		3.74 (0.19)		3.25 (0.13)		10.46 (0.51)	
>15%–25%	129	3.09 (0.15)		3.88 (0.33)		3.15 (0.21)		9.38 (0.66)	
>25%	140	3.29 (0.17)		4.46 (0.34)		2.97 (0.14)		8.49 (0.71)	
ECs			0.009		0.06		0.82		0.69
<5%	515	2.77 (0.08)		3.63 (0.15)		3.04 (0.08)		9.46 (0.38)	
5%–15%	288	3.05 (0.11)		3.65 (0.21)		3.28 (0.15)		10.49 (0.56)	
>15%–25%	98	3.04 (0.17)		3.96 (0.36)		3.34 (0.28)		9.31 (0.67)	
>25%	148	3.28 (0.17)		4.49 (0.37)		3.02 (0.13)		8.74 (0.73)	
WBCs			0.69		0.79		0.62		0.58
<5%	239	2.97 (0.11)		3.83 (0.20)		3.12 (0.13)		9.10 (0.47)	
5%–15%	387	2.91 (0.09)		3.53 (0.17)		3.15 (0.10)		10.44 (0.48)	
>15%–25%	185	2.88 (0.12)		3.92 (0.26)		3.03 (0.15)		9.28 (0.54)	
>25%	238	3.03 (0.13)		4.05 (0.27)		3.19 (0.15)		9.11 (0.54)	

a From test of linear trend.

## Discussion

DED is a complex heterogeneous condition with a wide array of symptoms, which makes its diagnosis and assessment of severity clinically challenging. Although a number of clinical tests have been developed to measure signs, most methods are limited by their subjective nature. Furthermore, these tests show low and inconsistent associations with patient-reported symptoms. Objectively measurable signs, including biomarkers that better correlate with symptoms, are much needed. In this regard, HLA-DR has been widely used in a number of studies to estimate severity of DED and monitor patient improvement and treatment response.^17^

In this study, we analyzed the expression pattern of HLA-DR in conjunctival cell populations from 1049 samples collected from 527 patients. This is the largest reported cohort of conjunctival samples included in a single DED study. On average, 15,507 ± 8673 cells were harvested from samples, with a range of 1061 to 88,097. Analyzable cells (>1000) were obtained from all but 5 samples (<0.5%), due in part to the extensive training provided to the clinical site personnel and strict adherence to study SOP. Samples with <10,000 cells (30% of our samples) were not excluded from analysis, as has been done by some studies.^20^,^30^,^46^,^47^ Previous studies in our lab and the data obtained in our present study after exclusion of samples with <10,000 cells support the inclusion of low cell number samples. Our data emphasize the importance of SOPs for sample collection and processing in multicenter clinical trials to maximize the number of analyzable patient samples and the number of tests/validations that can be performed.

The analysis of the two main constituents of conjunctival IC samples, EpCAM-expressing ECs and CD45-expressing WBCs, showed that 81.2% of the total gated cell population constituted ECs and 1.5% of WBCs, numbers consistent with previous observation.^19^ When we assessed the HLA-DR% positive cells in our samples, we found that on average 13% of the TCs expressed HLA-DR. Of the samples analyzed, 42.1% had <5% HLA-DR% in the TCs analyzed. Although these numbers are consistent with our group's previously reported data,^25^,^39^ the average % of HLA-DR positive cells is lower than values reported in other studies.^17^ Brignole-Baudouin et al.^23^ recently report a median of 75.68% HLA-DR%, with a range of 58.31%–86.1% in DE patients. Although the variation in average HLA-DR% between other studies of DED could be attributed to differences in inclusion criteria, the biggest contributor is likely the lack of standardized methods for flow cytometry instrumentation, data acquisition, and analysis. This has been acknowledged as an important issue in diseases where immunophenotyping is used for clinical diagnosis, the detection of rare phenotypes, and monitoring treatment response.^48^,^49^ Efforts to standardize sampling, flow cytometer performance between runs, and equalization of data obtained from different flow cytometers are already under way for other diseases; the European Union-supported EuroFlow Consortium and Human Immune Phenotyping Consortium are notable examples.^50^,^51^ Although our study did not involve multiple sites for sample processing and multiple flow cytometers for analysis, samples were acquired from 27 clinical sites. This was made possible by SOPs with stringent controls to reduce site-to-site variations in sample collection, storage temperature, and storage duration before processing. SOPs were also put in place to ensure comparable flow cytometry performance between analyses.

In addition to estimating % of HLA-DR% in the total population, we also determined the % of HLA-DR% by ECs and WBCs, an analysis done by very few other studies.^19^,^29^,^52^ As ECs constitute a high percentage of the TCs analyzed, the average % of HLA-DR-expressing ECs, at 12.5%, was close to the average of 13.0% for TCs. However, the average % of HLA-DR+ WBCs was, however, higher at 19.0%. A recent study has reported similar percentages of HLA-DR-expressing CD45+ cells in IC samples from DE patients.^29^ Although WBCs, at 1.5%, constituted a small percentage of the TC population obtained by IC, it is not surprising that on average a higher percentage of WBCs were positive for HLA-DR as APCs among the WBC population constitutively express HLA-DR.

Correlation analysis between HLA-DR% and symptoms and signs showed very few associations. A statistically significant correlation was only observed between HLA-DR% in both TCs and ECs with conjunctival staining scores. A significant correlation was also observed between HLA-DR% in TCs and corneal staining scores. In both observations, the mean conjunctival and corneal staining score increased with increasing levels of HLA-DR%. However, our observations regarding the correlation of HLA-DR% with symptoms and signs also varied from previous findings. Two studies with smaller sample sizes (<25 patients) have shown a statistically significant correlation with osmolarity (*r* = 0.614, *P* < 0.0001),^34^ with TBUT (*r* = −0.66, *P* = 0.0001), and with Schirmer's test (*r* = −0.62, *P* = 0.0001).^30^ The only study with a sample size (*n* = 311) comparable to our study demonstrated correlation with both symptoms and signs.^23^ In this study, which was a cumulative retrospective analysis of three clinical trials (SICCANOVE, SANSIKA, and NOSIKA), a correlation of HLA-DR% was observed with corneal staining (*r* = 0.26, *P* = 0.0001) and OSDI scores (*r* = 0.13, *P* = 0.03). Interestingly, when the same analysis was performed with mean fluorescence intensity of HLA-DR%, considered by the authors to better differentiate between samples that have reached the maximum threshold of HLA-DR%, significant associations with many signs and symptoms were observed. This included corneal staining (*r* = 0.30, *P* < 0.0001), TBUT (*r* = −0.13, *P* = 0.0226), Schirmer's test (*r* = −0.20, *P =* 0.0003), and OSDI (*r* = 0.12, *P* = 0.0426). However, the use of mean fluorescence intensity as a measurement parameter is controversial, especially in populations with a bimodal distribution pattern, as is observed with HLA-DR% by conjunctival cells (Fig. 1). Furthermore, unlike our study, IC sampling and correlation analysis with signs in this study were done for TCs from the “worst eye” based on higher scores for corneal fluorescein staining (CFS), lissamine staining, and combined CFS/lissamine staining for SICCANOVE, SANSIKA, and NOSIKA, respectively. It is also noteworthy that, although their average baseline scores for OSDI, CFS, and TBUT at 48.91 ± 22.5, 3.95 ± 2.06, and 3.07 ± 0.2, respectively, were not far from the present study's average scores (Table 2), their average Schirmer's test score at 4.55 ± 3.06 mm was much lower than that observed in the present study at 9.6 ± 7 mm. However, these authors collected samples without the administration of topical anesthetics. Studies have shown that wetting lengths for Schirmer's test done without anesthesia are significantly higher than those with anesthesia,^53^ raising the possibility of a more severe DE patient population as compared to our study. We also do not have a basis for comparison of our observations with conjunctival staining with the above described studies, as these studies, although including conjunctival lissamine staining score as an inclusion criteria, have not analyzed its correlation with HLA-DR%. Considering that conjunctival IC samples primarily consist of ECs, correlation of HLA-DR% in ECs with conjunctival and corneal staining scores is a significant finding and should be an important consideration for future studies. Lissamine is a vital dye that selectively stains compromised ECs, with positive conjunctival staining appearing in the early stages of DED.^54^ This explains why the strongest correlation seen is epithelial HLA-DR% and lissamine green staining. Changes in conjunctival EC HLA-DR% may, therefore, correlate with conjunctival staining scores when other signs may not.

Further investigation into the characteristics of the patient group with the highest HLA-DR% in conjunction with high conjunctival staining scores is needed to determine if the criteria can be used to identify/classify patient groups. Interestingly, the association of HLA-DR alleles with susceptibility to Sjogren's syndrome has been reported in several studies.^55^–^57^ Further studies might help determine if conjunctival HLA-DR% may serve as an additional criteria for the diagnosis of DED patient subgroups with Sjogren's syndrome.

In conclusion, there was a wide range of HLA-DR% in conjunctival cells of DREAM patients, which was not unexpected considering the heterogeneity of the study population and disease state. However, it is noteworthy that almost 42% of the study patients had <5% of conjunctival cells expressing HLA-DR at baseline. Thus, conjunctival HLA-DR% is not a sensitive marker of DED and may be of limited use to grade the severity of all DED patients. This is not surprising considering that DED is a multifactorial condition, with current standards of clinical diagnosis providing no clear delineations between different DE subtypes. However, HLA-DR% levels could prove useful in defining subtypes of DED patients prone to epithelial disease, such as those with higher levels of ocular surface staining.
